# Gaps and Barriers Among Dental Undergraduates Towards Promoting and Assisting Tobacco Cessation: A Multicountry Cross‐Sectional Study

**DOI:** 10.1155/ijod/3216074

**Published:** 2026-03-06

**Authors:** Divya Gopinath, Nurul Hanis Ramzi, P. V. K. S. Hettiarachchi, Fawaz Pullishery, Irna Sufiawati, Anupam Podder, Shermin Hashir, Ashish Shrestha

**Affiliations:** ^1^ Basic Medical and Dental Sciences Department, Ajman University, P. O. Box 346, Ajman, UAE, ajman.ac.ae; ^2^ Centre of Medical and Bio-allied Health Sciences Research, Ajman University, Ajman, UAE, ajman.ac.ae; ^3^ Centre for Cancer and Stem Cell Research, Institute for Research, Development and Innovation, IMU University, Kuala Lumpur, 57000, Malaysia, imu.edu.my; ^4^ Department of Oral Medicine and Periodontology, Faculty of Dental Sciences, University of Peradeniya, Kandy, Sri Lanka, pdn.ac.lk; ^5^ Center for Research in Oral Cancer, Faculty of Dental Sciences, University of Peradeniya, Kandy, Sri Lanka, pdn.ac.lk; ^6^ Division of Dental Public Health, Faculty of Dentistry, Batterjee Medical College, Jeddah, Saudi Arabia, bmc.edu.sa; ^7^ Department of Oral Medicine, Faculty of Dentistry, Padjadjaran University, Sumedang, Indonesia, unpad.ac.id; ^8^ Department of Periodontology and Oral Pathology, Dhaka Dental College, Dhakha, Bangladesh; ^9^ College of Dental Medicine, Department of Preventive and Restorative Dentistry, University of Sharjah, Sharjah, UAE, sharjah.ac.ae; ^10^ Department of Oral Pathology, College of Dental Surgery, BP Koirala Institute of Health Sciences, Dharan, Nepal, bpkihs.edu

**Keywords:** awareness, barriers, dental students, dentist, tobacco cessation

## Abstract

The purpose of this study was to assess and compare awareness, preparedness, and perceived barriers to promoting and assisting tobacco cessation among dental students in South/South East Asia. This cross‐sectional study of dental undergraduate students undergoing clinical training at seven participating dental schools across seven countries used an online questionnaire to assess three main attributes: awareness, preparedness, and perceived barriers to promoting and assisting with tobacco cessation. A total of 667 undergraduate dental students participated in the study. Though the students demonstrated good theoretical knowledge and willingness to engage in tobacco cessation practices, 60.7% perceived, they lacked sufficient practical experience to provide tobacco cessation counseling (TCC), and 27.7% reported, they lacked sufficient clinical time. Approximately one‐third of the students (31.3%) expressed concern that the dentist–patient relationship would be affected if they insisted on providing TCC. Further, 64.8% also perceived patient‐related barriers as a lack of motivation to quit tobacco, whereas 26.3% and 21.9% of students believed that patients were not expecting or unwilling to listen to a dental student, respectively. A moderate positive relationship was observed between awareness and preparedness (*r* = 0.583, *p*  < 0.001), suggesting that enhanced knowledge improves confidence in counseling. Conversely, the negative correlations between awareness and barriers (*r* = −0.317, *p*  < 0.001) and between preparedness and barriers (*r* = −0.365, *p*  < 0.001) indicated that better training can help overcome implementation challenges. Although the dental students believed they had reasonable awareness and willingness, several gaps were identified, especially in translating this knowledge to practice in dental clinics across different countries.

## 1. Introduction

According to the 2019 statistics by the World Health Organization (WHO), there are about 1.337 billion tobacco users worldwide, and more than 8 million people are reported to die because of tobacco‐induced diseases annually [1]. Smoking and smokeless tobacco constitute the two primary forms of tobacco use. Tobacco consumption will remain a significant public health threat in the coming decades [2]. Asia is the world’s largest producer and consumer of tobacco [3]. It has been reported that 90 % of smokeless tobacco users are from South and Southeast Asian countries [4].

The harmful effects of smoking tobacco on the human body and its different systems are comprehensively studied. Smoking exposes the oral tissues to several toxic chemicals, including carcinogens. Smokers have been known to show 5–10 times higher risk for oral cancer compared to nonsmokers [5]. Tobacco smoke is a significant determinant in the prognosis of periodontal diseases and implant treatment, while also contributing to oral hard tissue discoloration, restoration staining, and halitosis [6]. Smokeless tobacco, which is very popular in South and Southeast Asian countries, has been implicated in causing numerous mucosal pathologies, including oral cancer and pre‐cancers, according to the type, frequency, and duration of use [6, 7]. The risk attributable to smokeless tobacco for oral cancer varies widely across the globe, ranging from 1.36 to 7.90 [8]. Smokeless tobacco has also been associated with diseases of the teeth and their supporting structures, including gingival recession, gingival attachment loss, root exposure, and dental caries [9]. Healthcare professionals widely use tobacco cessation counseling (TCC) to increase abstinence rates in smokers and smokeless tobacco workers [10]. With most tobacco users seeking dental visits frequently, dental health professionals have a unique opportunity to support patients in abstaining from tobacco use [11]. The recent Cochrane review has reported that tobacco cessation interventions by oral health professionals in dental settings might increase the abstinence rates among both smokers and smokeless tobacco users [12–14]. Dental professionals possess the cognitive skills and competence to recognize oral health manifestations associated with tobacco use and can, thus, convey the message more effectively [15, 16]. Dental treatment often includes multiple appointments and, therefore, serves as an ideal opportunity for patient support by initiating and reinforcing tobacco cessation protocols.

Moreover, dental appointments present a unique opportunity to use oral health conditions as feedback on the detrimental effects of tobacco on oral and general health. Several studies have identified that dentists tend to score well in the knowledge aspect of tobacco cessation [17]. However, accumulated evidence highlights the substantial gaps in oral health professionals’ engagement in tobacco cessation practices. Dentists are less involved in actively promoting tobacco cessation than other health professionals, despite the evident need for such an effort [18–20].

Most dental and medical schools often deliver the basics of tobacco education in their curricula [12, 21]. Tobacco cessation policies are included in dental coursework in the United States; however, effective integration into the curriculum has not yet been achieved, and tobacco cessation practices are not components of many dental schools [13, 14, 22]. Even though faculties know tobacco‐related pathology, interest, and skills are often lacking to supervise the integration of this practice in regular patient care in the undergraduate curriculum, or they have limited formal training amongst themselves [14, 22, 23]. A European workshop has also proposed guidelines for introducing tobacco cessation policies into predoctoral curricula [24, 25]. Although the implementation of tobacco cessation practices in dental curricula has been advocated by many dental organizations worldwide, detailed, comparative data on curricular content, instructional hours, clinical assessment methods, and the application of standardized frameworks within Southeast Asian institutions remain and underdocumented.

Despite the strong evidence as well as an urge from various dental organizations to support tobacco cessation, the problem persists as to why the desired outcome still needs to be achieved. Few isolated studies have reported positive attitudes in the students towards tobacco cessation practices [26, 27]. However, understanding future dental health professionals’ preparedness and perceived barriers is a significant step toward understanding the challenges involved in the appropriate operationalization and holistic integration of tobacco cessation practices into the curriculum and routine dental clinical care. Such information, collected using a standardized tool, could help compare results across various settings. It could have important implications for policy and guideline recommendations, particularly for implementing/modifying tobacco cessation training in the dental curriculum. This can help enhance future dentists’ involvement in tobacco cessation in dental school clinical training as well as independently in their future dental practice, ultimately leading to a decline in tobacco use. The objective of this study was to assess and compare awareness, preparedness, and perceived barriers to promoting and assisting tobacco cessation among dental students from different countries in South/South East Asia.

## 2. Methods

**Figure 1:**
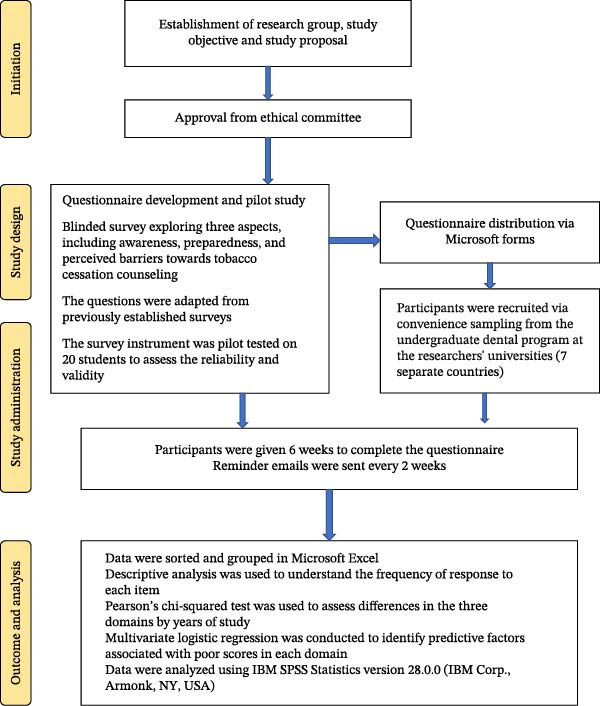
Flow chart for research methodology.

This study was conducted as a cross‐sectional study from December 2021 to May 2022. This study was approved by the International Medical University Joint Research Committee under Project Number 16/JCM‐222/2021. The approval was also obtained from the relevant bodies of the participating institutions. The study was conducted in accordance with the ethical standards of the institutional ethics committee and the guidelines of the 1964 Declaration of Helsinki. Informed consent was obtained from all participants. The dental students included in the study were from Sri Lanka, Indonesia, Malaysia, Nepal, Bangladesh, Saudi Arabia, and the UAE. The only inclusion criterion was that participants be dental undergraduate students in their clinical training years (Years 3, 4, and 5) and willing to participate in the study. The research methodology is graphically represented in Figure 1.

### 2.1. Sample Size and Sampling Methodology

To estimate the sample size required to detect a difference in each domain towards promoting tobacco cessation between dental students in different countries of South/Southeast Asia, the equation of *N* = 2 (*Z*_*α*/2_ + *Z_β_*)^2^ × 2 × *σ* ^2^/*d* ^2^ was used with 80% power at the 0.05 level of significance [28–30]. We expected the population standard deviation to be 1.0 (*σ* = 1). Keeping *α* = 0.05 and *β* = 0.2 (80% power), *Z_α_*_/2_ = 1.960, *Z*_*β*_ = 0.8416, *σ* = 1, and *d* = 0.25, the minimum sample size required is calculated as 502 [29, 30]. However, given the possibility of incomplete questionnaires, a 10% loss is expected; thus, the adjusted sample size was calculated to be 557. All the dental undergraduate students undergoing clinical training (undergraduates from the third year onwards) were invited to participate in this study from participating dental schools in seven South/South East Asian countries. Consequently, participant enrollment followed a convenience sampling approach.

### 2.2. Data Collection

The data were collected via an anonymous online survey in English using Microsoft Forms. Translation of the survey was deemed unnecessary because the participant pool was composed exclusively of dentistry students with English‐medium instruction. The online survey invitation included all information about the study, as well as details on the confidentiality of the survey data. The online survey questionnaire was adapted from established scales used in earlier studies by Liu et al. [31] and Murugaboopathy et al. [26]. The questionnaire was pilot‐tested on 20 students to assess its reliability and validity. The analysis yielded a Cronbach’s alpha coefficient of 0.82, indicating good internal consistency. Informal verbal feedback from students who completed the pilot study questionnaire confirmed its clarity, relevance, and internal consistency. Consequently, it was deemed suitable for use in the main study without any revisions. To avoid bias, participants in the pilot study were excluded from the main sample. The questionnaire comprised three main attributes: awareness, preparedness, and perceived barriers towards promoting and assisting in tobacco cessation. The questionnaire consisted of 25 questions with a five‐point Likert response scale, ranging from strongly disagree to strongly agree. The questionnaire also included demographic data, including country of origin, ethnicity, gender, year of dental school, and tobacco use history, which were recorded as nonusers, former users, or current users. The survey was open for 6 weeks, and reminder emails were sent to students every 2 weeks.

### 2.3. Data Analysis

The Likert scale responses were coded as 1–5, from strongly disagree to strongly agree, following the guidelines of the previous study by Liu et al. [31], and entered into Microsoft Excel. A descriptive analysis of response frequency was performed. Pearson’s chi‐squared test (*χ* ^2^) was used to assess differences in awareness, preparedness, and perceived barriers to practicing TCC, as measured by questionnaire items, by years of study among dental students from South/Southeast Asian countries. The independent variables included awareness, preparedness, and perceived barriers towards practicing TCC. Scores for these domains were calculated by summing the relevant items and classified into the following thresholds: poor (<60%), fair (61%–75%), and good (>75%). A multivariable logistic regression analysis was conducted to identify predictive factors associated with poor scores across the three domains of TCC. The relationships between the three domains, awareness, preparedness, and perceived barriers were assessed using Pearson’s correlation coefficient. Data was analyzed using IBM Statistical Package for the Social Sciences version 28.0.0 (SPSS Inc., Chicago, IL, USA).

## 3. Results

The study sample comprised 667 undergraduate dental students; 23.2% (*n* = 155) were males. The nonresponse rate was 42% in total. The study sample was collected from seven countries. Indonesian dental students comprised about one‐quarter of the sample (39.3%), while the rest were as follows: UAE (10.6%), Sri Lanka (18.4%), Saudi Arabia (6.6%), Nepal (15.1%), Malaysia (6%), and Bangladesh (3.9%). Complete demographic data are described in Table S1.

### 3.1. Students’ Awareness, Preparedness, and Perceived Barriers Towards Practicing TCC

The distribution of dental students’ awareness, preparedness, and perceived barriers to practicing tobacco cessation, as measured on a five‐point Likert scale, is shown in Table 1. Almost all respondents (93.8%) reported understanding the role of tobacco in the etiology of oral cancer. In addition, 64.3% and 66.4% of students knew about TCC and were eligible to practice the TCC protocol, respectively. Most dental students (91.7%) reported they would advise patients to quit tobacco use in their future career life, and 87.3% were confident in explaining the negative impacts of tobacco use on oral health. Although nearly half of the students (48.3%) knew the mechanism of action of nicotine replacement therapy (NRT), only 31.9% and 18.2% knew they were eligible to prescribe NRT and the NRT dosage, respectively. Almost half of the students always recorded the amount of tobacco their patients used over the years (56.5%) or made an effort to assist a patient in quitting tobacco use more than once (58.7%).

**Table 1:** Distribution of dental students’ awareness, preparedness, and perceived barriers towards practicing tobacco cessation counseling.

Item	Strongly disagree	Disagree	Neutral	Agree	Strongly agree
Awareness					
I know what tobacco cessation protocol is (*n* = 667)	16 (2.4)	47 (7.0)	175 (26.2)	297 (44.5)	132 (19.8)
I am aware of the “5A’s” and the “5R’s” protocol of tobacco cessation counseling (*n* = 666)	41 (6.1)	118 (17.7)	161 (24.1)	223 (33.4)	123 (18.4)
I know that I am eligible to practice tobacco cessation (*n* = 664)	24 (3.6)	32 (4.8)	167 (25.2)	282 (42.5)	159 (23.9)
I understand tobacco has a role in etiology of oral cancer and poor periodontal outcomes (*n* = 666)	12 (1.8)	3 (0.5)	26 (3.9)	172 (25.8)	453 (68.0)
I know the mechanism of action of nicotine replacement therapy (*n* = 666)	29 (4.4)	114 (17.1)	201 (30.2)	236 (35.4)	86 (12.9)
I know I am eligible to prescribe nicotine replacement therapy (*n* = 664)	44 (6.6)	127 (19.1)	281 (42.3)	162 (24.4)	50 (7.5)
I know the dosage of nicotine replacement therapy (*n* = 666)	81 (12.2)	243 (36.5)	221 (33.2)	90 (13.5)	31 (4.7)
Preparedness					
I will advise patients to quit tobacco use in my future career (*n* = 665)	12 (1.8)	11 (1.7)	32 (4.8)	223 (33.5)	387 (58.2)
I believe tobacco cessation counseling provided by dentists could assist patients to quit tobacco use (*n* = 664)	8 (1.2)	15 (2.3)	71 (10.7)	324 (48.8)	246 (37.0)
I am confident in explaining the negative impacts of tobacco usage on oral health (*n* = 662)	8 (1.2)	9 (1.4)	67 (10.1)	313 (47.3)	265 (40.0)
I always record the amount of tobacco my patients used over the years (e.g., pack year) in patient folder (*n* = 662)	19 (2.9)	61 (9.2)	208 (31.4)	245 (37.0)	129 (19.5)
I always record the type of tobacco in patient folder (e.g., cigarettes, cigars, and e‐cigarettes) (*n* = 662)	19 (2.8)	61 (9.1)	203 (30.4)	253 (37.9)	126 (18.9)
I have made effort to assist a patient to quit tobacco use more than once (*n* = 662)	21 (3.1)	52 (7.8)	197 (29.5)	275 (41.2)	117 (17.5)
Perceived barriers					
I cannot accurately determine patient’s’ smoking history without being intrusive (*n* = 661)	27 (4.1)	112 (16.9)	322 (48.7)	182 (27.5)	18 (2.7)
I do not consider tobacco cessation counseling part of the dentist’s professional role (*n* = 661)	182 (27.5)	320 (48.0)	100 (15.0)	38 (5.7)	21 (3.1)
Giving smoking cessation counseling is not part of my role as a dental student (*n* = 661)	224 (33.9)	298 (45.1)	85 (12.9)	36 (5.4)	18 (2.7)
Patients do not expect tobacco cessation counseling from a dental student (*n* = 662)	66 (10.0)	206 (31.1)	216 (32.6)	154 (23.3)	20 (3.0)
Patients do not listen to dental students during tobacco cessation counseling (*n* = 662)	55 (8.3)	225 (34.0)	237 (35.8)	120 (18.1)	25 (3.8)
I am concerned that the tobacco cessation message may alienate tobacco‐using patients (*n* = 659)	27 (4.1)	131 (19.9)	295 (44.8)	181 (27.5)	25 (3.8)
Giving unwanted tobacco cessation counseling may upset the dentist‐patient relationship (*n* = 662)	42 (6.3)	170 (25.7)	219 (33.1)	210 (31.7)	21 (3.2)
Many tobacco‐using patients do not have the motivation to quit (*n* = 659)	16 (2.4)	68 (10.3)	148 (22.5)	340 (51.6)	87 (13.2)
Self‐motivation is the most important factor for a patient to quit (*n* = 663)	13 (2.0)	7 (1.1)	59 (8.9)	219 (33.0)	365 (55.1)
Tobacco cessation counseling is ineffective unless the patient has a related health problem (*n* = 662)	67 (10.1)	185 (27.9)	179 (27.0)	189 (28.5)	42 (6.3)
Clinical time is limited so I tend to focus on dental treatments instead of counseling (*n* = 661)	39 (5.9)	213 (32.2)	226 (34.2)	150 (22.7)	33 (5.0)
There is no tobacco cessation information (e.g., leaflets or pamphlets) available in the hospital which I can distribute to my patient (*n* = 660)	28 (4.2)	124 (18.8)	219 (33.2)	225 (34.1)	64 (9.7)
There is no referral pathway for tobacco‐using patients in my institution (*n* = 659)	33 (5.0)	136 (20.6)	260 (39.5)	180 (27.3)	50 (7.6)
I do not have the enough theoretical knowledge (*n* = 661)	40 (6.1)	212 (32.1)	214 (32.4)	179 (27.1)	16 (2.4)
I do not have the enough practical experience in tobacco cessation counseling (*n* = 660)	21 (3.2)	66 (10.0)	172 (26.1)	305 (46.2)	96 (14.5)

*Note:* Data are presented with frequency and percentage of students’ response on the Likert scale for each item on the questionnaire, *n* (%).

Students also reported several barriers to the delivery of TCC. About sixty‐one percent of the undergraduates (60.7%) perceived not having enough practical experience to provide TCC, and 27.7% of students reported not having enough clinical time to introduce TCC to the patients. Approximately one‐third of the postgraduates (31.3%) had concerns about the dentist–patient relationship if they insisted on providing TCC to their patients. In addition, the students reported a lack of tobacco cessation information in the dental hospital (43.8%) and were unaware of a referral pathway (34.9%). Patient‐related barriers included: (1) they do not have the motivation to quit tobacco use (64.8%), (2) they are not expecting TCC from a dental student (26.3%), (3) they are not listening to TCC from a dental student (21.9%), and (4) they will consider tobacco cessation only when they have a related health problem (34.8%). Almost all students (85.8%) believed that TCC provided by dentists could help patients quit tobacco.

### 3.2. Variations in Awareness, Preparedness, and Perceived Barriers by Study Years Among Dental Undergraduates

The proportion of “Agree” and “Strongly agree” responses by study years is presented in Table 2. Significant differences were observed between most awareness domain items and study years among the dental students, such that 64.3% and 51.9% of the students knew and were aware of the TCC protocol and the “5A’s” and “5R’s” protocol, respectively (both *p*  < 0.001). In addition, almost half of the students knew the mechanism of NRT regardless of study year (48.6%, *p*  < 0.001); however, few knew they were eligible to prescribe NRT (31.6%, *p* = 0.003) or the NRT dosage (18.0%, *p* = 0.02). For the preparedness domain, there are significant differences between study years for students who consistently record the amount of tobacco their patients used over the years (56.1%, *p*  < 0.001), record the type of tobacco in patient folder (57.0%, *p*  < 0.001) and made an effort to assist a patient in quitting tobacco use more than once (59.0%, *p*  < 0.001). Only two items in the perceived barriers domain have shown significant differences across study years, with 64.3% of dental students reporting that many tobacco‐using patients do not have the motivation to quit (*p* = 0.01) and 43.7% reporting the non availabaility of tobacco cessation information (e.g., leaflets or pamphlets) was available at the hospital (*p*  < 0.001).

**Table 2:** Variations of awareness, preparedness, and perceived barriers towards practicing tobacco cessation counseling among multinational dental students.

Item	Year 3	Year 4	Year 5	Years 3–5	*p*‐Value
	Proportion (%) of “agree” and “strongly agree”				
Awareness					
I know what tobacco cessation protocol is	**123/227 (54.2%)**	**147/210 (70.0%)**	**155/224 (69.2%)**	**425/661 (64.3%)**	**<0.001**
I am aware of the “5A’s” and the “5R’s” protocol of tobacco cessation counseling	**81/227 (35.7%**	**126/210 (60.0%)**	**136/224 (60.7)**	**343/661 (51.9%)**	**<0.001**
I know that I am eligible to practice tobacco cessation	141/227 (62.1%)	145/210 (69.0%)	151/224 (67.4%)	437/661 (66.1%)	0.27
I understand tobacco has a role in etiology of oral cancer and poor periodontal outcomes	214/227 (94.3%)	198/210 (94.3%)	207/224 (92.4%)	619/661 (93.6%)	0.65
I know the mechanism of action of nicotine replacement therapy	**87/227 (38.3%)**	**122/210 (41.9%)**	**112/224 (50.0%)**	**321/661 (48.6%)**	**<0.001**
I know I am eligible to prescribe nicotine replacement therapy	**57/227 (25.1%)**	**75/210 (35.7%)**	**77/224 (34.4%)**	**209/661 (31.6%)**	**0.03**
I know the dosage of nicotine replacement therapy	**29/227 (12.8%)**	**38/210 (18.1%)**	**52/224 (23.2%)**	**119/661 (18.0%)**	**0.02**
Preparedness					
I will advise patients to quit tobacco use in my future career	207/227 (91.2%)	193/210 (91.9%)	205/224 (91.5%)	605/661 (91.5%)	0.97
I believe tobacco cessation counseling provided by dentists could assist patients to quit tobacco use	194/227 (85.5%)	181/210 (86.2%)	190/224 (84.8%)	565/661 (85.5%)	0.92
I am confident in explaining the negative impacts of tobacco usage on oral health	196/227 (86.3%)	185/210 (88.1%)	192/224 (85.7%)	573/661 (86.7%)	0.75
I always record the amount of tobacco my patients used over the years (e.g., pack year) in patient folder	**104/227 (45.8%)**	**142/210 (67.6%)**	**125/224 (55.8%)**	**371/661 (56.1%)**	**<0.001**
I always record the type of tobacco in patient folder (e.g., cigarettes, cigars, and e‐cigarettes)	**107/227 (47.1%)**	**141/210 (67.1%)**	**129/224 (57.6%)**	**377/661 (57.0%)**	**<0.001**
I have made effort to assist a patient to quit tobacco use more than once (*n* = 662)	**109/227 (48.0%)**	**142/210 (67.6%)**	**139/224 (62.1%)**	**390/661 (59.0%)**	**<0.001**
Perceived barriers					
I cannot accurately determine patients’ smoking history without being intrusive	72/227 (31.7%)	62/210 (29.5%)	66/224 (29.5%)	200/661 (30.3%)	0.84
I do not consider tobacco cessation counseling part of the dentist’s professional role	21/227 (9.3%)	19/210 (9.0%)	19/224 (8.5%)	59/661 (8.9%)	0.96
Giving smoking cessation counseling is not part of my role as a dental student	22/227 (9.7%)	12/210 (5.7%)	20/224 (8.9%)	54/661 (8.2%)	0.28
Patients do not expect tobacco cessation counseling from a dental student	52/227 (22.9%)	68/210 (32.4%)	53/224 (23.7%)	173/661 (26.2%)	0.05
Patients do not listen to dental students during tobacco cessation counseling	44/227 (19.4%)	48/210 (22.9%)	53/224 (23.7%)	145/661 (21.9%)	0.51
I am concerned that the tobacco cessation message may alienate tobacco‐using patients	75/227 (33.0%)	66/210 (31.4%)	64/224 (28.6%)	205/661 (31.0%)	0.58
Giving unwanted tobacco cessation counseling may upset the dentist‐patient relationship	77/227 (33.9%)	65/210 (31.0%)	89/224 (39.7%)	231/661 (34.9%)	0.15
Many tobacco‐using patients do not have the motivation to quit	**137/227 (60.4%)**	**127/210 (60.5%)**	**161/224 (71.9%)**	**425/661 (64.3%)**	**0.01**
Self‐motivation is the most important factor for a patient to quit	197/227 (86.8%)	188/210 (89.5%)	196/224 (87.5%)	581/661 (87.9%)	0.66
Tobacco cessation counseling is ineffective unless the patient has a related health problem	81/227 (35.7%)	72/210 (34.3%)	77/224 (34.4%)	230/661 (34.8%)	0.94
Clinical time is limited so I tend to focus on dental treatments instead of counseling	55/227 (24.2%)	59/210 (28.1%)	69/224 (30.8%)	183/661 (27.7%)	0.29
There is no tobacco cessation information (e.g., leaflets or pamphlets) available in the hospital which I can distribute to my patient	**76/227 (33.5%)**	**108/210 (51.4%)**	**105/224 (46.9%)**	**289/661 (43.7%)**	**<0.001**
There is no referral pathway for tobacco‐using patients in my institution	67/227 (29.5%)	83/210 (39.5%)	80/224 (35.7%)	230/661 (34.8%)	0.08
I do not have the enough theoretical knowledge	67/227 (29.5%)	65/210 (31.0%)	62/224 (27.7%)	194/661 (29.3%)	0.75
I do not have the enough practical experience in tobacco cessation counseling	136/227 (59.9%)	126/210 (60.0%)	137/224 (61.2%)	399/661 (60.4%)	0.96

*Note:* Data are presented with frequency and percentage of “positive” students’ response by study years. Statistically significant of proportion variations between the questionnaire items and years of study are shown in bold.

### 3.3. Predictive Factors for Poor Domains in Practicing TCC

#### 3.3.1. Awareness

In examining factors associated with poor awareness in practicing TCC, dental students from the UAE and Indonesia showed statistically significantly lower odds compared to students from other countries. The academic year also played a role, with third‐year students showing a lower likelihood of poor awareness (higher awareness; OR = 0.38, 95% CI: 0.21–0.72, *p* = 0.003) than fifth‐year students. Gender, tobacco use, and tobacco use by immediate family members do not show significant associations with poor awareness in this context (Table 3).

**Table 3:** Multivariate logistic regression to analyze the predictive factors for poor domains towards practicing tobacco cessation counseling.

Predictive factors	Poor awareness [OR (95% CI)]	*p*‐Value	Poor preparedness [OR (95% CI)]	*p*‐Value	Poor perceived barriers [OR (95% CI)]	*p*‐Value
Country						
UAE	0.102 (0.020–0.521)	0.006	0.510 (0.092–2.221)	0.440	2.056 (0.177–23.893)	0.565
Sri Lanka	0.286 (0.058–1.415)	0.125	0.233 (0.044–1.654)	0.076	1.530 (0.750–6.833)	0.657
Saudi Arabia	0.265 (0.043–1.622)	0.151	0.326 (0.063–1.086)	0.098	10.250 (0.903–116.291)	0.060
Nepal	0.520 (0.099–2.727)	0.440	1.062 (0.200–5.639)	0.943	1.265 (0.135–11.892)	0.837
Malaysia	0.213 (0.034–1.333)	0.098	0.767 (0.118–5.009)	0.782	4.143 (0.425–40.338)	0.221
Indonesia	0.206 (0.043–0.986)	0.048	1.324 (0.234–7.487)	0.751	2.395 (0.281–20.407)	0.424
Bangladesh	Reference					
Academic year						
3rd year	0.385 (0.205–0.721)	0.003	1.324 (0.234–7.487)	0.751	1.271 (0.526–3.068)	0.594
4th year	1.584 (0.787–3.189)	0.198	1.138 (0.177–7.314)	0.892	1.129 (0.424–3.004)	0.808
5th year	Reference					
Gender						
Male	1.192 (0.649–2.189)	0.560	0.473 (0.163–1.859)	0.810	3.253 (1.477–7.163)	0.003
Female	Reference					
Tobacco use						
Nonuser	0.271 (0.030–2.445)	0.244	0.461 (0.059–1.718)	0.097	0.460 (0.088–2.405)	0.358
Current user	0.231 (0.022–2.451)	0.224	0.161 (0.025–1.604)	0.142	0.913 (0.122–6.819)	0.929
Previous user	Reference					
Tobacco use by immediate family						
Nonuser	1.328 (0.580–3.043)	0.502	1.267 (0.297–3.116)	0.210	1.367 (0.397–4.706)	0.620
Current user	1.310 (0.512–3.355)	0.574	0.731 (0.231–3.011)	0.624	0.673 (0.163–2.785)	0.585
Previous user	Reference					

#### 3.3.2. Preparedness

Regarding poor preparedness, country of origin and academic year reveal some differences, though most are not statistically significant. Students from Saudi Arabia had a lower OR (0.33; 95% CI: 0.06–1.09; *p* = 0.098) than those from other countries, but this difference was not statistically significant. Third‐ and fourth‐year students both showed slightly higher odds of poor preparedness, though these differences are not substantial. Gender, tobacco use, and immediate family tobacco use similarly do not show significant associations with poor preparedness, indicating a limited impact of these variables on preparedness levels among students (Table 3).

#### 3.3.3. Perceived Barriers

Factors associated with poor (low) perceived barriers show notable associations, particularly gender. Male students are significantly less likely to report perceived barriers than females, with an OR of 3.25 (95% CI: 1.47–7.16, *p*  < 0.001). Country of origin, academic year, and tobacco use do not show significant relationships with perceived barriers, suggesting that gender is a primary predictor in this domain (Table 3).

### 3.4. Correlation Between Awareness, Preparedness, Perceived Barriers, and Study Years Among Dental Undergraduates

The correlation analysis among awareness, preparedness, and perceived barriers in practicing TCC is shown in Table 4. A moderate positive correlation (*r* = 0.583, *p*  < 0.001) exists between awareness and preparedness, indicating that higher awareness is associated with greater preparedness for counseling. At the same time, awareness is significantly negatively correlated with perceived barriers (*r* = −0.317, *p*  < 0.001), suggesting that greater awareness is associated with fewer perceived barriers. Similarly, preparedness is negatively correlated with perceived barriers (*r* = −0.365, *p*  < 0.001), suggesting that greater preparedness is associated with fewer barriers in counseling.

**Table 4:** Correlation between three domains for practicing tobacco cessation counseling.

Domains	Awareness	Preparedness	Perceived barriers
Awareness			
Pearson correlation	–	0.583	−0.317
*p*‐Value		0.000	0.000
Preparedness			
Pearson correlation	–	–	−0.365
*p*‐Value			0.000

## 4. Discussion

The WHO recommends that dentists and other oral health professionals assist patients in quitting smoking and integrate tobacco cessation into dental practice. We evaluated the attitude of dental undergraduates who are also future dental professionals toward tobacco cessation.

Our results suggest that most dental undergraduates in this study held positive attitudes and views toward the dental professional’s role in managing tobacco users, regardless of the Asian country in which they were. Almost all the students believed that TCC provided by dentists could help patients quit tobacco. This high level is consistent with another recent study from Hong Kong [31], which found that most dental undergraduates believed that TCC is a part of their role as dentists and would advise patients to quit smoking later in their careers. This is also consistent with previous studies conducted across other countries in the earlier decade [32–36], in which the majority of students showed a positive attitude towards tobacco cessation. A previous study from US reported that fourth‐year students were more likely to consider TCC within the dental scope, a finding not influenced by gender or personal and family tobacco histories [37]. However, we did not find any statistical difference in attitude across years. In contrast, another study from Nigeria reported that dental students underestimated the importance of dental professionals in assisting tobacco counseling and did not believe it could be effective in helping patients quit tobacco use [38]. This could be possibly due to a lack of training and resources to carry out the TCC in dental schools and clinics.

There were differences in knowledge of TCC across countries; 50%–60% of students possessed a theoretical understanding of the 5As and 5Rs of tobacco cessation. A significant proportion of the students lacked the knowledge needed to prescribe NRT. Even if patients were motivated by tobacco cessation, dentists might require knowledge of pharmacotherapy products to provide meaningful assistance. The most recent Cochrane review suggests that there is currently insufficient evidence to conclude that providing behavioral support alone from dental professionals increases tobacco cessation rates [39]. There is evidence with a moderate degree of certainty that tobacco abstinence rates among smokers rise when dental professionals provide medication along with behavioral support. To be assured of the extent of the benefit and whether combining pharmacological therapies is more beneficial than behavioral assistance alone, more research is needed. Hence, pharmacotherapy can be considered the way forward, and dentists need to be trained in these approaches.

Dental curricula must emphasize to students the importance of acquiring the skills for proactive, upstream approaches to protect against the harmful oral health effects of tobacco, rather than reactive, downstream approaches that treat tobacco effects on the hard and soft tissues of the oral cavity. Motivating patients to quit smoking is a skill that dental students can develop through proper techniques, such as motivational interviewing [40, 41]. However, 31% of students expressed concerns about the dentist–patient relationship if dentists insisted on providing TCC to their patients, compared with 41% in another study [31]. Our survey further revealed that 43.8% of undergraduates perceived a lack of cessation information in their hospital, and 34.9% were unaware of a referral pathway, rates lower than those reported by their counterparts in Hong Kong (58% and 38%, respectively [31]. Similar to previous studies [21, 26, 27], almost all undergraduates (85.8%) believed that TCC provided by dentists could help patients quit tobacco. However, the undergraduates perceived a lack of motivation among patients to discontinue tobacco use as the main patient‐related barrier (64.8%), followed by a willingness to quit only when they have a health‐related problem 34.8%. In addition, a minority, 26.3% and 21.9% perceived that the patients were not expecting TCC from a dental student and were not listening to TCC from a dental student, respectively.

Our study revealed a significant gap regarding the clinical translation of this knowledge in clinical practice. The most critical reported barrier was that the students needed more practical experience to provide TCC. This could be because teaching on the relationship between tobacco use and oral health remains at a knowledge‐based level, and more emphasis is placed on the impact of tobacco use on oral health. A survey of Australian dental students revealed that teaching about the risks of tobacco use in the etiology of oral cancer was prioritized over teaching about smoking cessation counseling [36]. Another European study report also stated that dental schools followed a similar pattern, with only 40% reporting providing practical training [41]. To address this disparity, dental schools should emphasize reinforcing the practical aspect of TCC training. Problem‐based learning sessions or task‐based learning on using the TCC protocol and NRT effectively in clinical practice can be implemented in the curriculum. Another option would be incorporating smoking cessation into the patient’s clinical treatment plan. Hence, students would be motivated to complete the treatment. Further, it also highlights the necessity for national guidelines on oral health protection that explicitly integrate tobacco cessation as a core competency. Such guidelines would provide a consistent framework for dental curricula, clinical protocols, and public health messaging, ensuring that future dental professionals are equipped to address tobacco use systematically and effectively.

Although our international study contributes to the growing literature on the preparedness of dentists for tobacco cessation, it has some limitations that warrant consideration when interpreting the findings. As students who are particularly interested in tobacco cessation are more likely to participate and complete the survey, the use of convenience sampling and online student surveys is a limitation that could have led to an overestimation of the variables of knowledge, positive attitudes, and practices. Further the data were collected at a single point in time, and hence this study cannot track the evolution of attitudes and behaviors without longitudinal follow‐up. The use of closed‐ended questions in our survey may have forced responses into predetermined categories that did not fully capture the complexity of participants’ opinions or experiences. The lack of open‐ended questions limited the ability to gather rich, qualitative data. Despite pilot testing, some survey items may have been ambiguous, misunderstood, or interpreted differently by various subgroups of respondents, leading to measurement error. The study’s results are sufficiently generalizable to most South and Southeast Asian nations with comparable educational backgrounds, but are not generalizable to other parts of the world.

## 5. Conclusion

This multicountry study reveals that while students possess adequate theoretical knowledge and positive attitudes toward TCC, they lack the practical training and clinical confidence to implement it effectively. The significant proportion of students reporting insufficient practical experience and clinical time coupled with perceived patient‐related and relational barriers, underscores a systemic failure to translate awareness into clinical competency. To address this, dental curricula must move beyond theoretical inclusion and adopt standardized, competency‐based TCC modules that include behavioral counseling techniques, cultural communication skills, and clear clinical referral pathways. Ultimately, national dental accreditation bodies should mandate TCC as a core clinical competency, ensuring future dentists are equipped not merely as oral surgeons, but as essential front line advocates in the broader public health mission to reduce tobacco‐related morbidity and mortality.

## Author Contributions

Conceptualization, validation, resources, project administration: Divya Gopinath. Methodology, data curation, writing – original draft preparation, funding acquisition: Divya Gopinath and Nurul Hanis Ramzi. Software, formal analysis: Nurul Hanis Ramzi. Investigation, writing – review and editing: Divya Gopinath, P. V. K. S. Hettiarachchi, Shermin Hashir, Fawaz Pullishery, Irna Sufiawati, Ashish Shrestha, and Anupam Podder.

## Funding

The APC is funded by Ajman University.

## Disclosure

All authors have read and agreed to the published version of the manuscript. The authors take full responsibility of the content of the manuscript.

## Ethics Statement

This study was approved by the International Medical University Joint Research Committee under Project Number 16/JCM‐222/2021.

## Conflicts of Interest

The authors declare no conflicts of interest.

## Supporting Information

Additional supporting information can be found online in the Supporting Information section.

## Supporting information


**Supporting Information** Students’ demographic profile (*n* = 667).

## Data Availability

The data are available upon request from authors.
