# Supplementary material for: Gaps and Barriers Among Dental Undergraduates Towards Promoting and Assisting Tobacco Cessation: A Multicountry Cross‐Sectional Study
Source: Int J Dent. 2026 Mar 6;2026:3216074. doi: 10.1155/ijod/3216074 (PMC12964073; doi:10.1155/ijod/3216074)
Supplement: Supplementary file 1 — Supporting Information Students’ demographic profile (n = 667). [file IJOD-2026-3216074-s001.docx]

| **Table 1. Students’ demographic profile (n= 667).** | | | | | | | |
| --- | --- | --- | --- | --- | --- | --- | --- |
|  | |  | |  | |  |  |
| Demographic Profile | | | | | n (%) | |  |
| Year of study^#^ | | | | |  |  | |
|  | Year 3 | | | |  | 227 (34.03) | |
|  | Year 4 | | | |  | 210 (31.48) | |
|  | Year 5 | | | |  | 224 (33.58) | |
| Country |  | | | |  |  | |
|  | United Arab Emirates | | | |  | 71 (10.6) | |
|  | Sri Lanka | | | |  | 123 (18.4) | |
|  | Saudi Arabia | | | | | 44 (6.6) | |
|  | Nepal | | | |  | 101 (15.1) | |
|  | Malaysia | | | |  | 40 (6.0) | |
|  | Indonesia | | | | | 262 (39.3) | |
|  | Bangladesh | | | | | 26 (3.9) | |
| Sex* |  | | | |  |  | |
|  | Male | | | |  | 155 (23.2) | |
|  | Female | | | |  | 510 (76.5) | |
| Student tobacco use status* | | | | | | | |
|  | Non-user | | | | | 613 (91.9) | |
|  | Current user | | | | | 35 (5.2) | |
|  | Former user | | | | | 17 (2.5) | |
| Family members’ tobacco use status^¥^ | | | | | | | |
|  | Non-user | | | | | 445 (66.7) | |
|  | Current user | | | | | 154 (23.1) | |
|  | Former user | | | | | 67 (10.0) |  |
| ^#^n=661 | | |  |  |  |  |  |
| *n=665 | | |  |  |  |  |  |
| ^¥^n=666 | | |  |  |  |  |  |
|  | | |  |  |  |  |  |
